# 295. Methods for Cost-efficient Real-time Whole Genome Sequencing Surveillance for Enhanced Detection of Outbreaks in a Hospital Setting

**DOI:** 10.1093/ofid/ofae631.085

**Published:** 2025-01-29

**Authors:** Kady D Waggle, Marrisa P Griffifth, Alecia B Rokes, Alexander Sundermann, Vaughn Cooper, Lee Harrison, Lora Pless

**Affiliations:** University of Pittsburgh, Pittsburgh, PA; University of Pittsburgh, Pittsburgh, PA; University of Pittsburgh, Pittsburgh, PA; University of Pittsburgh, Pittsburgh, PA; University of Pittsburgh School of Medicine, Pittsburgh, Pennsylvania; University of Pittsburgh, Pittsburgh, PA; University of Pittsburgh, Pittsburgh, PA

## Abstract

**Background:**

Healthcare associated infections (HAI) are common hospital complications associated with increased length of stay, economic burden, morbidity, and mortality. Traditionally, HAI outbreaks were detected using reactive whole genome sequencing (WGS); however, with costs decreasing over time, WGS has become a practical tool for the real time detection of nosocomial outbreaks. This study describes such methods, provides a cost analysis, and reviews notable outbreaks observed while performing real time WGS surveillance of HAIs.

EDS-HAT real-time genomic surveillance methods

**Figure 1:**
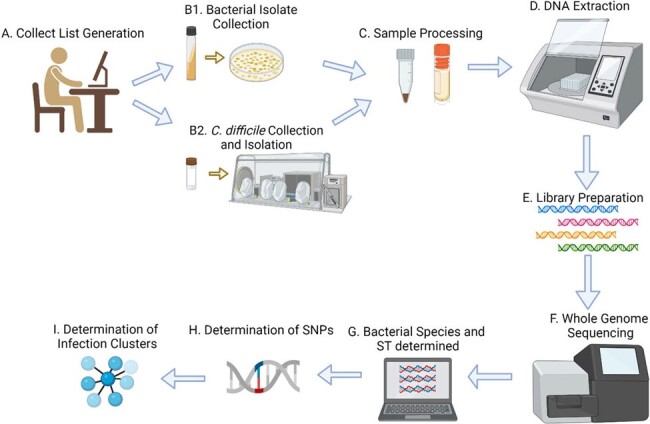
EDS-HAT real-time genomic surveillance methods A) collect list generation, B) sample collection C) sample processing, D) DNA extraction, E) Library preparation, F) WGS, and G-I) Data analysis, including determining SNPs between samples and infection clusters

**Methods:**

The Enhanced Detection System for Healthcare-Associated Transmission (EDS-HAT) was devised to identify potential transmitted HAIs in real time among patients admitted to the hospital for ≥ 3 days or with a previous exposure in the prior 30-days (Fig 1). We performed a cost analysis for real time genomic HAI surveillance, based on an average cutoff of 35× coverage per genome, considering personnel, reagents, supplies, and WGS platform. We also considered maximum sample counts that could be sequenced on two platforms (MiSeq or NextSeq), based on genome size (Fig 2). Bioinformatic analyses were completed following WGS, and epidemiological links were investigated for isolates that clustered with ≤ 15 pair-wise SNP differences (*C. diff* ≤ 2 SNPs).

Maximum Sample Counts

**Figure 2:**
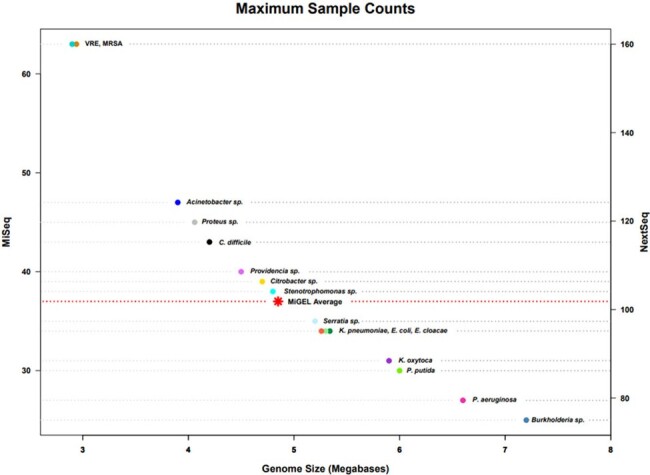
Maximum number of genomes that can be run on a MiSeq v3 600 cycle flow cell and NextSeq500 v2.5 300 cycle flow cell based on size (Mb). Sample counts were calculated using Illumina coverage calculator based on 80× coverage criteria. Genome size of an average run by the MiGEL lab is shown in comparison

**Results:**

From November 1, 2021 to October 31, 2023, we sequenced 4,723 isolates (weekly mean N=49). The per sample cost depended on platform used and sample count, totaling $49 or $19 for MiSeq (N=32) and NextSeq (N=96), respectively (Table 1). With the inclusion of personnel costs for 1 lab technician and 1 biostatistician (salary only, based on percent effort) the average weekly cost to run EDS-HAT was $4,293 (range $3,626-$4,758). We highlight multiple outbreaks involving a common hospital unit, equipment, or location (Table 2). Following Infection Prevention (IP) intervention, no additional cases were detected for these clusters on the associated routes.

Cost per sample Breakdown

**Table 1.**
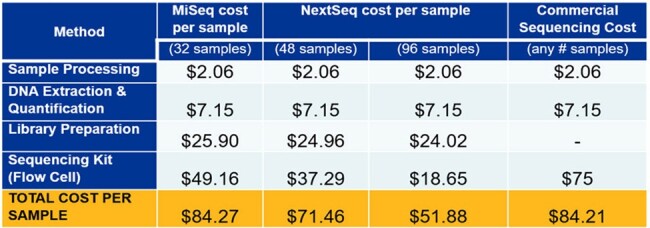
Cost per sample across MiSeq, NextSeq, and commercial WGS

**Conclusion:**

We describe time- and cost-efficient methods for real time outbreak surveillance and detection using WGS. These genomic methods, as described in EDS-HAT, can be utilized with traditional epidemiological methods to guide hospital IP teams to mitigate and stop the spread of deadly transmitted HAIs.

Notable Outbreaks

**Table 2.**
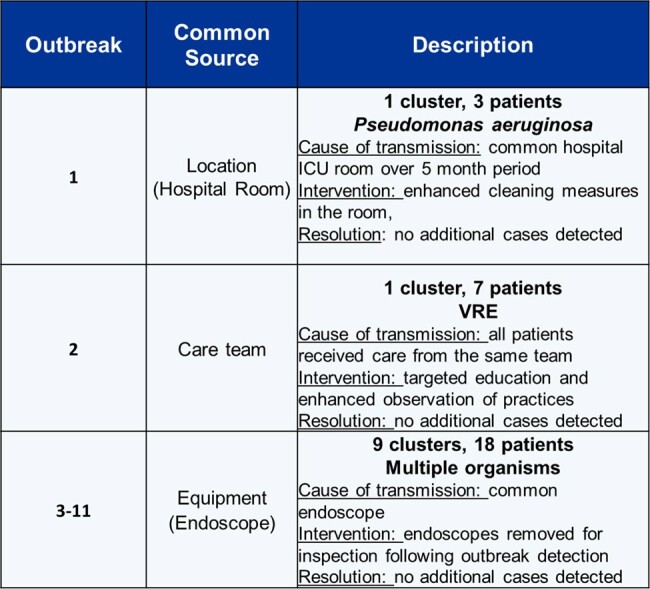
Description of notable outbreaks detected by real-time EDS-HAT HAI surveillance

**Disclosures:**

**Alexander Sundermann, DrPH, CIC, FAPIC**, OpGen: Honoraria **Lee Harrison, MD**, GSK: Advisor/Consultant|Merck: Advisor/Consultant|Pfizer: Advisor/Consultant|Sanofi: Advisor/Consultant

